# Observations on emergence of mannitol-use-deficient *Staphylococcus aureus*

**DOI:** 10.1128/spectrum.03091-25

**Published:** 2025-12-19

**Authors:** Patrick M. Schlievert, Samuel H. Kilgore, Takeshi Yoshida, Lisa A. Beck, Donald Y. M. Leung

**Affiliations:** 1 Department of Microbiology and Immunology, Carver College of Medicine, University of Iowa 4083, Iowa City, Iowa, USA; 2 Department of Dermatology, University of Rochester Medical Center 6923, Rochester, New York, USA; 3 Department of Pediatrics, National Jewish Health 2930, Denver, Colorado, USA; University of Guelph College of Biological Science, Guelph, Ontario, Canada

**Keywords:** *Staphylococcus aureus*, mannitol deficient, clone, atopic dermatitis

## Abstract

*Staphylococcus aureus* clones emerge and recede in roughly 10-year intervals unless virulence factors impose clonal continuation. This study investigates a newly emergent clone. Prior to 2020, all of 322 clinical isolates tested grew, as expected, yellow colonies on mannitol salt agar. After 2020, 10% of clinical *S. aureus* isolates grew as intensely pink colonies (hyper-pink, not pale red as seen in coagulase-negative staphylococci). The hyper-pink *S. aureus* lacked the gene mannitol-1-phosphate dehydrogenase within the mannitol fermentation operon. The organisms were methicillin-sensitive. *S. aureus* usually expresses cell-surface virulence factors during exponential growth and secretes toxins for a brief time in the post-exponential phase, but not in the stationary phase. The hyper-pink organisms expressed both cell-surface and secreted virulence factors well into the stationary phase. *S. aureus*, which is usually yellow on mannitol salt, showed the virulence factor production profile of hyper-pink organisms when the media pH was maintained at 8.0. Comparison of hyper-pink organism culture fluids with those of USA300 and USA400 *S. aureus strains* for interaction with epithelial cells revealed similar harmful pro-inflammatory properties. Known global regulatory systems in the hyper-pink organisms were intact, including the two-component system SrrA/B, which senses both oxygen and pH. This newly emergent clone appears to have been selected after 2020 by its inability to ferment mannitol. The clone was cultured from the skin of patients with moderate to severe atopic dermatitis.

**IMPORTANCE:**

A novel clone of *Staphylococcus aureus* emerged after 2020, unusually, with production of cell-surface and secreted virulence factors into late stationary growth phase. This newly emerging clone may be confused with coagulase-negative staphylococci because it has a strong pink phenotype on mannitol salt agar, whereas *S. aureus* usually forms bright yellow colonies on this medium.

## INTRODUCTION

There are many clonal groups of human *Staphylococcus aureus*, originally defined as bacteriophage types (or groups) and, more recently, classified by multiple methods, including pulsed-field gel electrophoresis (PFGE) groups USA100–USA1100. In the United States, bacteriophage types 29/52 emerged in hospitals in 1950, peaked in 1955, and caused nearly all serious *S. aureus* infections. This clone died back by 1960 but was replaced in the 1960s by a bacteriophage clone called 52/52A, peaking in 1965 and then falling back by 1970 (1–3). In 1971, a new clone arose, more recently referred to as USA200 (1–3). This latter clone did not die back (1–3), possibly because of the production of the superantigen toxic shock syndrome toxin-1 (TSST-1) (4), giving the organisms a selective survival advantage in humans. This clone remains common today. In the 1990s, a new clone again arose, a methicillin-resistant *S. aureus* (MRSA) capable of causing fatal hemorrhagic pneumonia (USA400) (5, 6). These organisms produced the superantigens staphylococcal enterotoxin B (SEB) or staphylococcal enterotoxin C (SEC) (7). This clone remains common in the Midwest of the United States. Finally, in 2000, another clone arose, this time also being MRSA and causing hemorrhagic pneumonia, referred to as USA300 (8, 9). In this manuscript, we describe a new clone of *S. aureus*, which arose after 2020 in atopic dermatitis patients.

The most straightforward method to identify *S. aureus* from skin and mucous membranes, where the organisms usually reside in colonized and infected humans, is to culture swabs on mannitol salt agar. This medium contains mannitol, which can be fermented by *S. aureus* to yield acid end products that change the pH indicator phenol red from pale red to bright yellow (10). The medium also contains sodium chloride, with staphylococci being halo-tolerant (as opposed to most other bacteria). Thus, coagulase-positive *S. aureus* organisms turn the media bright yellow after 24 to 48 h of growth, coagulase-negative opportunistic staphylococci grow but retain the red color of the media, and other bacteria do not grow. Recently, we began observing and isolating a group of hyper-pink colonies growing on mannitol salt agar plates. These would usually be considered variants of coagulase-negative, opportunistic staphylococci. However, when we examined the organisms, they were coagulase-positive *S. aureus*. Phenotypic and genetic characteristics of these “new” colony types are also addressed in this manuscript.

The virulence of *S. aureus* is defined by production of both cell-surface virulence factors, such as protein A, during the exponential growth phase, and secreted toxin virulence factors, including cytotoxins and superantigens, just before the organisms enter stationary phase (*in vitro* usually in the 1-hour time period of 5 × 10^8^ to 5 × 10^9^ colony-forming units (CFUs)/mL) (11). Virulence factor production does not continue into the late stationary phase (11).

The production of virulence factors by *S. aureus* is regulated by multiple operons and two-component systems (TCSs). Among these, the TCS staphylococcal respiratory response (Srr)A/B is required for secreted toxin virulence factor production (12, 13). Growth of *S. aureus* in the presence of oxygen and pH 7.5–8.0 is required (14), while *S. aureus* may grow anaerobically. It is thought that the SrrA/B sensing system is recognizing redox potential (12, 13, 15, 16); thus, acidic pH may reflect fermentation occurring in the absence of oxygen utilization. Another important global regulator is the accessory gene regulatory (*agr*) operon and TCS (11). Agr is required for cell-surface virulence factor production during the exponential phase and secreted toxin production in post-exponential phase, but only when the medium is oxygenated and pH is neutral or higher (11). These regulatory systems are also investigated in this study.

## RESULTS

**Fig 1:**
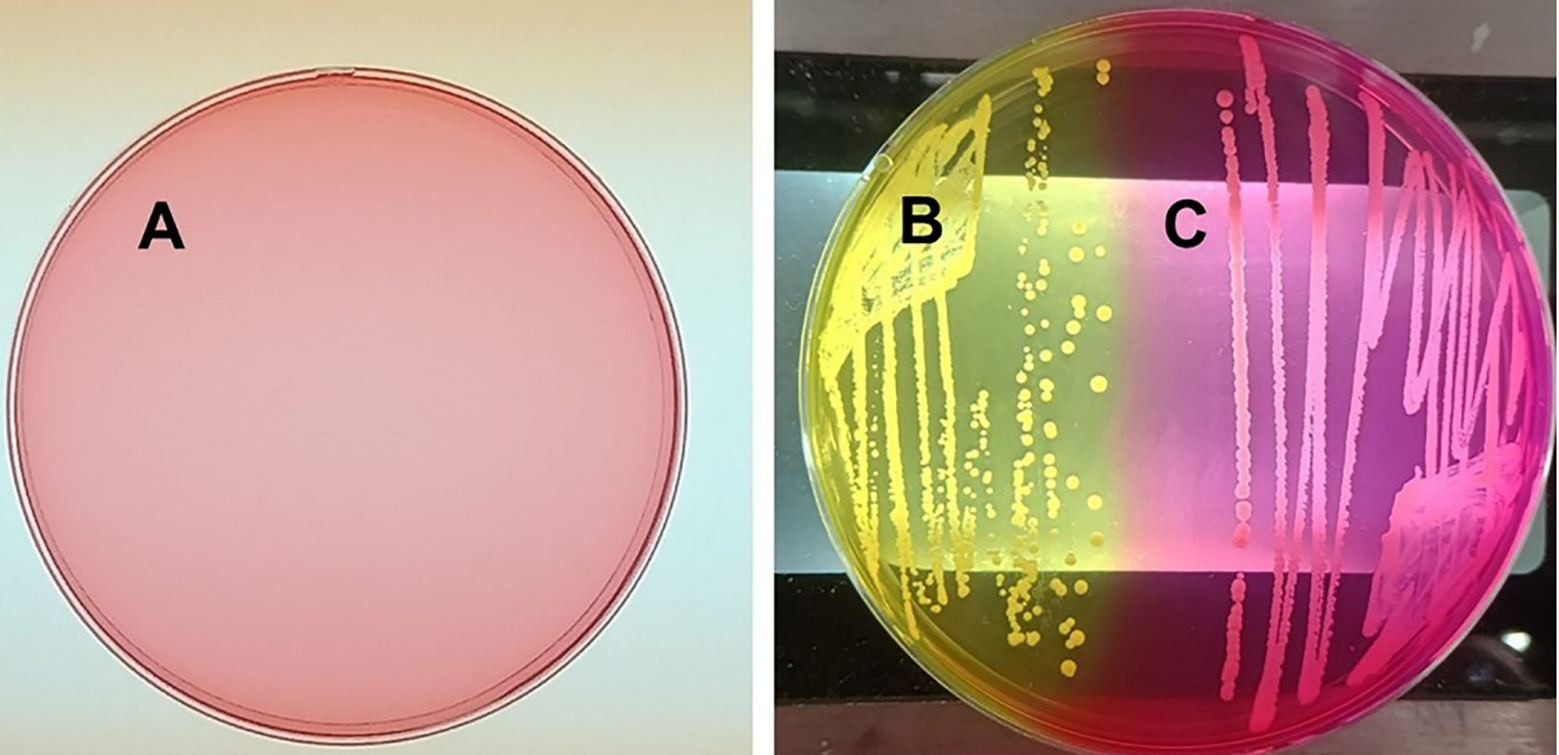
Appearance of hyper-pink *Staphylococcus aureus* on mannitol salt agar (MSA). (**A**) Uninoculated MSA. (**B**) Usual *S. aureus* on MSA. (**C**) Hyper-pink *S. aureus* on MSA.

In 2021, we began observing hyper-pink *S. aureus* on mannitol salt agar plates (Fig. 1). Mannitol salt agar plates have phenol red as the pH indicator, which is pale red when the medium is made (Fig. 1A). The typical *S. aureus* isolated prior to 2021 grew as bright yellow colonies after overnight incubation at 37°C due to acidification of the medium (Fig. 1B). The post-2020 emergent hyper-pink *S. aureus* grew as intensely pink colonies through oxidative metabolism, making the medium basic (Fig. 1C). The organisms shown in Fig. 1C may be mistaken as coagulase-negative staphylococci since the medium does not become yellow, even after extended incubation for one week.

Approximately 10% (12/125) of clinical *S. aureus* from multiple disease sources, including atopic dermatitis (AD) and toxic shock syndrome (TSS), were hyper-pink upon isolation from 2021 to present. The hyper-pink phenotype continued upon extensive subculturing. No yellow variants on any plates were observed. In contrast, no hyper-pink organisms were observed prior to 2021 with 322 atopic dermatitis and TSS isolates tested (*P* < 0.0001). None of the pre-2021 isolates showed hyper-pink mutants, even upon continuous subculturing. Clinical isolates from atopic dermatitis and TSS as far back as 1980, when we began receiving AD and TSS isolates, did not exhibit the hyper-pink phenotype.

**Fig 2:**
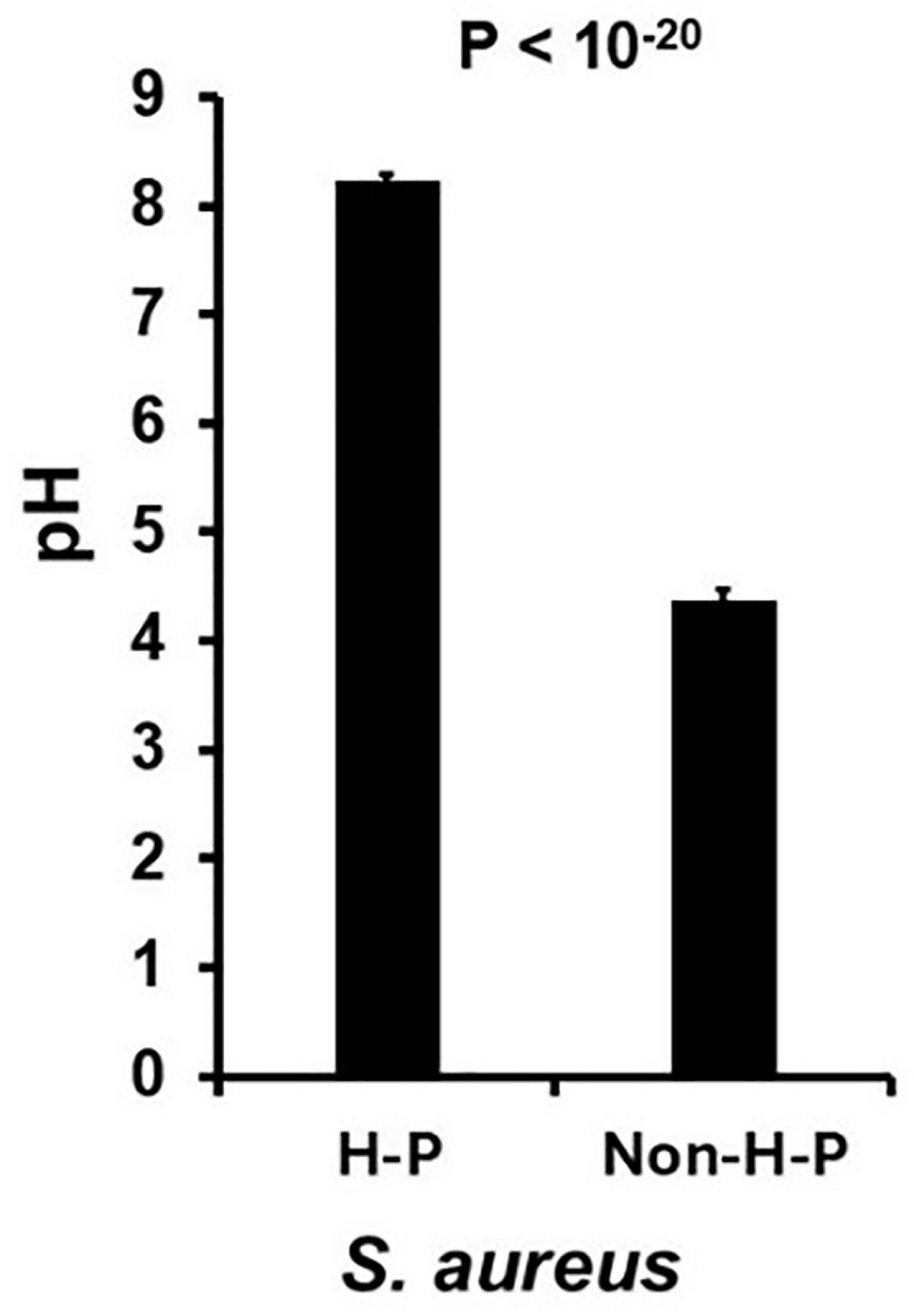
pH of mannitol salt agar plates after culture of hyper-pink (H-P) *S. aureus* versus non-hyper-pink (Non-H-P) *S. aureus*. Data from seven hyper-pink and eight non-hyper-pink are reported as means ± standard deviations, with Student’s *t*-test *P* value.

The intense color of the hyper-pink organisms was suggestive of oxidative metabolism on MSA plates, with the medium becoming basic. This was tested by measuring the pH of the mannitol salt plates after 2 days of *S. aureus* culture on MSA at 37°C. The pH of seven hyper-pink organisms after 2 days of incubation at 37°C was 8.24, compared to 4.38 for eight non-hyper-pink (typical) organisms (*P* < 10^−20^) (Fig. 2).

**Fig 3:**
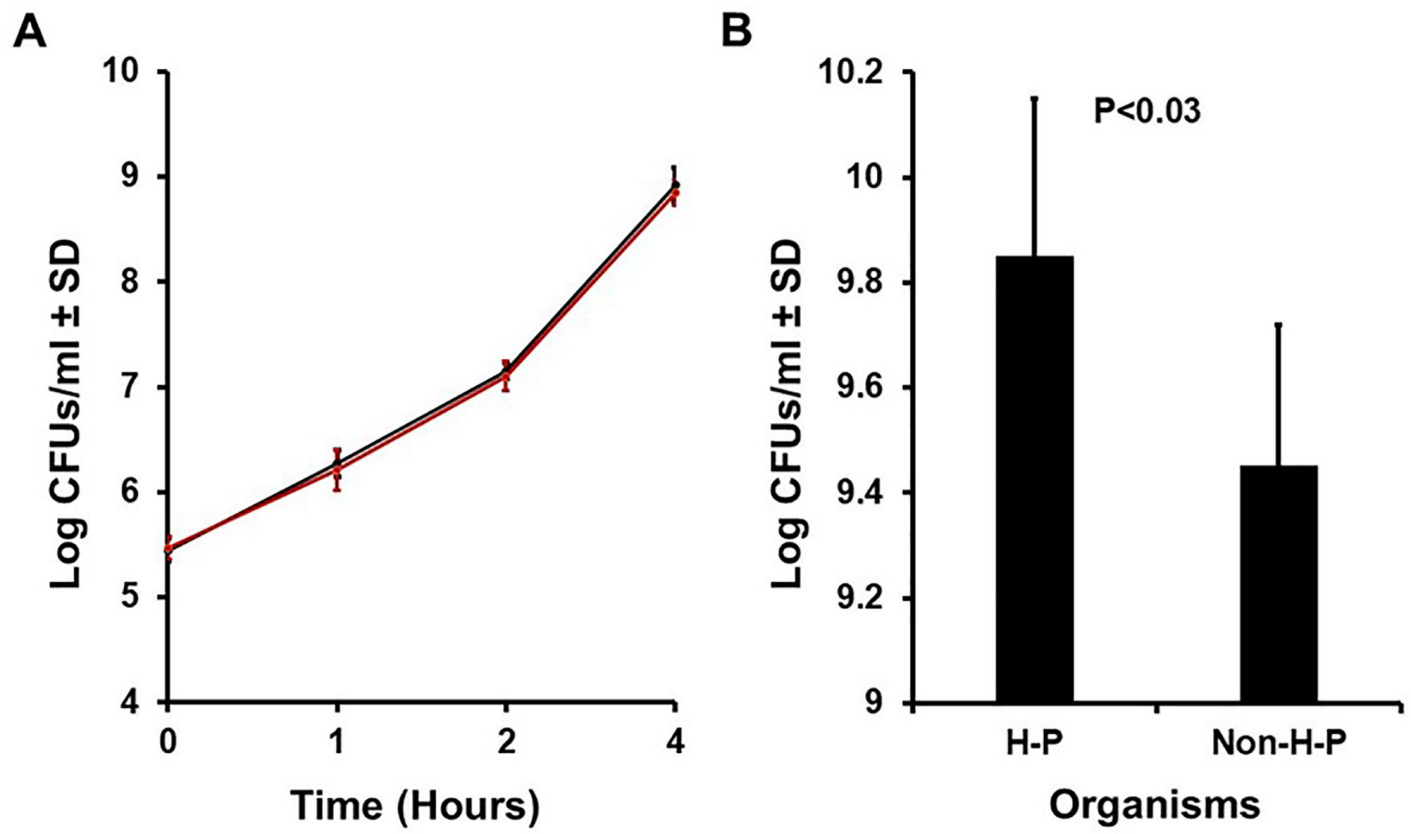
(**A**) Growth kinetics during exponential phase (average log_10_CFUs/mL; 0 to 4 h) at 37°C with 200 revolutions/min shaking of 7 hyper-pink *S. aureus ±* standard deviation (SD) (black line) compared to eight non-hyper-pink organisms ± SD (red line). (**B**) Mean stationary phase of growth (24 h) at 37°C and 200 revolutions/min shaking of seven hyper-pink (H-P) *S. aureus* ± SD compared to eight non-hyper-pink (Non-H-P) organisms ± SD.

The same seven hyper-pink strains and eight non-hyper-pink strains were then cultured in Todd Hewitt broths at 37°C with shaking (200 revolutions per minute) to evaluate growth kinetics (Fig. 3A) and stationary phase (Fig. 3B). Both groups generally had the same growth kinetics (Fig. 3A), but the hyper-pink organisms grew with somewhat higher CFUs/mL at stationary phase (Fig. 3B). The hyper-pink organisms averaged 8.74 × 10^9^/mL (log_10_ CFUs/mL = 9.85 ± 0.30 standard deviation) at stationary phase, compared to 3.30 × 10^9^/mL (log_10_ CFUs/mL = 9.45 ± 0.27 standard deviation) for the non-hyper-pink organisms (*P* < 0.03). Thus, it appeared that the hyper-pink *S. aureus* were better able to utilize the nutrients in the Todd Hewitt medium than the standard non-hyper-pink *S. aureus* organisms, consistent with the apparent primary oxidative metabolism of the hyper-pink organisms.

The seven hyper-pink organisms were methicillin-sensitive based on susceptibility to oxacillin using the disk method (17).

**Fig 4:**
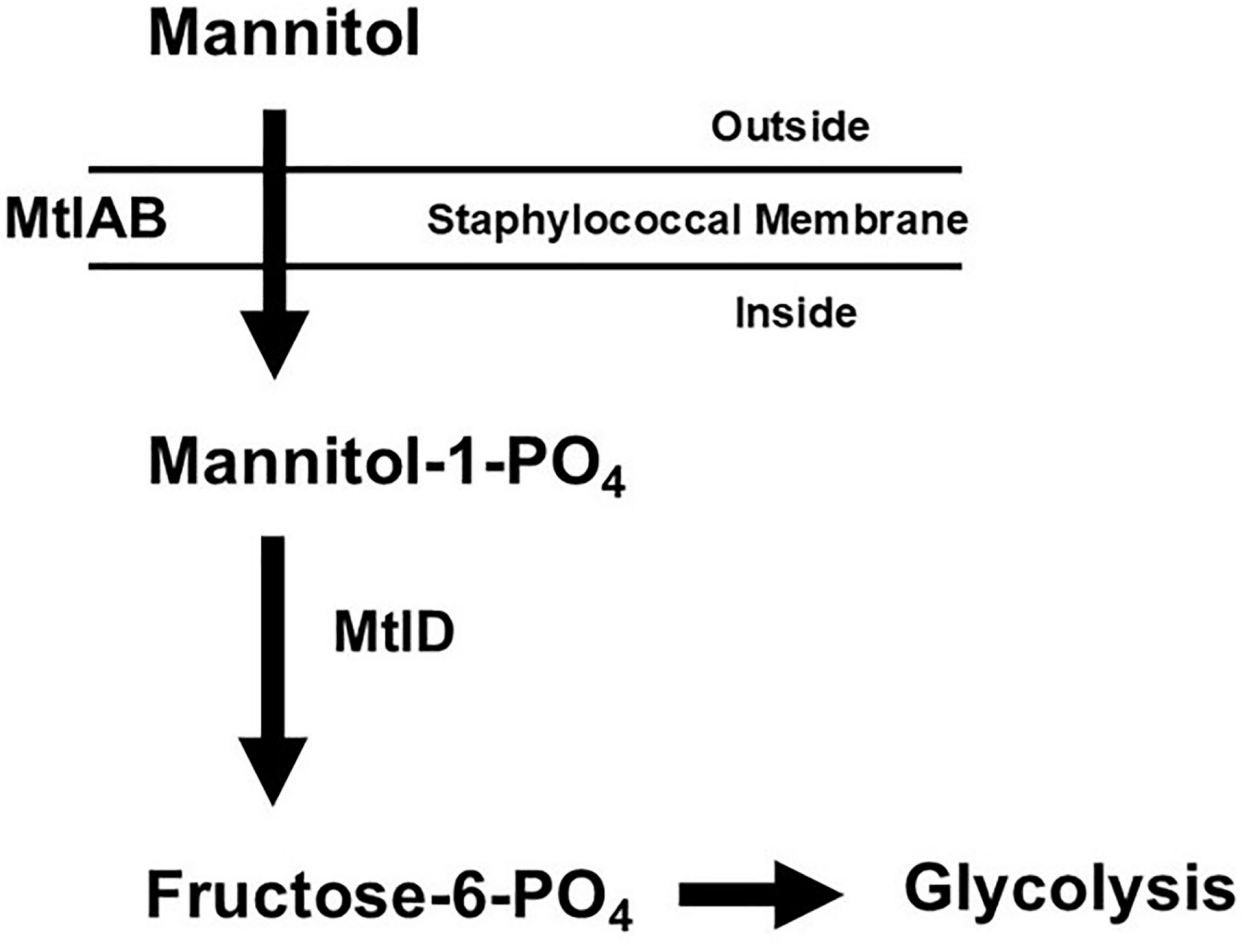
Mannitol (Mtl) utilization operon (*mtlABFD*) in *Staphylococcus aureus*. Mtl-1-phosphate-5-dehydrogenase, encoded by (*mtlD*)*,* catalyzes the conversion of mannitol-1-phosphate to fructose-6-phosphate, which then enters into the Embden-Meyerhoff and hexose-monophosphate glycolytic pathways (10).

**Fig 5:**
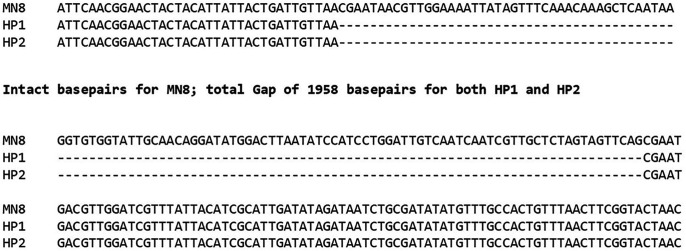
Sequence of a section of the mannitol operon, showing the deletion gap in the mannitol-1-phosphate 5-dehydrogenase gene in HP1 and HP2. The entire mannitol operon was present in *S. aureus* strain MN8, but there was the same 1958 base-pair deletion in both HP1 and HP2. HP1 and HP2 were from different patients seen at different geographic locations in the United States.

The first two hyper-pink organisms (HP1 and HP2), which we isolated post-2020, were sequenced at the Seq Center (Pittsburgh, PA) and compared with sequences of standard organisms isolated prior to 2021. Comparisons of major global regulatory DNA elements and the mannitol utilization pathway were made. At least two major global regulatory systems linked to virulence, SrrA/B and Agr, were intact. In contrast, the mannitol utilization pathway (Fig. 4) in the sequenced organisms each had the same large deletion mutation (Fig. 5) in mannitol-1-phosphate 5-dehydrogenase (*mtlD*), and the pathway was thus not expressed.

All seven tested hyper-pink isolates had the same or similar deletion, as determined by polymerase chain reaction using primers just outside the gene encoding mannitol-1-phosphate 5-dehydrogenase. It is thus likely that these organisms were clonal, having the same or similar mutation, even though the organisms were obtained from diverse parts of the United States.

We also tested the hyper-pink organisms HP1 and HP2 for the production of known *S. aureus* virulence factors. Both had intact genes for the cell-surface virulence factor protein A, the secreted cytotoxins α-toxin and phenol-soluble modulins, and the secreted superantigen staphylococcal enterotoxin-like X (SE*l*-X), which has been associated with pneumonia (18). Previously, we established that the pattern of superantigen production also predicts clonal groups (7, 19, 20). Thus, the production of SE*l*-X, in the absence of other major superantigens, adds strength to the clonality of these organisms. Additionally, the production of α-toxin and SE*l*-X in the absence of many other superantigens is suggestive of USA300 organisms (18, 21). However, the majority of the USA300 clonal group is MRSA (22), unlike the hyper-pink organisms.

Like *S*. *aureus*, *S. pseudintermedius* is coagulase-positive. Although the organism is more common in animals, it can be observed in humans. We examined HP1 and HP2 for their relatedness to the genomes of *S. intermedius*. HP1 and HP2 shared minimal sequence similarity to *S. intermedius* (14% of the genomes). In contrast, the entire HP1 and HP2 genomes were nearly 98% similar to other *S. aureus* genomes.

Secreted virulence factors are only produced by *S. aureus* in the presence of at least 3% oxygen (13), even though the organism can grow anaerobically, using fermentation metabolism instead of oxidative metabolism. SrrA/B is an important oxygen sensing TCS in *S. aureus* (12, 15, 16). However, it is unlikely that the histidine kinase component, SrrB, directly senses oxygen. It is more likely that SrrB is sensing redox potential in the membrane. The hypothesis is as follows: oxidative metabolism in *S. aureus* causes the pH of the growth medium to remain neutral to basic. Fermentation, including use of mannitol, causes the pH of the medium to become acidic. *S. aureus* produces exotoxins under oxidative pH conditions and not during fermentation, when pH is reduced to pH 6.0 or lower (14). Thus, *S. aureus* senses both pH and oxygen, suggesting that it can detect redox potential in the membrane (12, 23). Under usual conditions, exotoxins are thought to be produced *in vitro* only for approximately 1 h during post-exponential phase, and then, toxin production is stopped (11). The stoppage of exotoxin production in stationary phase correlates with reduced pH of the medium (24). In contrast, cell-surface virulence factors, such as protein A, are thought to be produced only during exponential phase and not produced during post-exponential and stationary phases (11). This differential regulation of secreted versus cell-surface virulence factors is thought to be important for early walling off of *S. aureus* infections (cell-surface dependent) versus later spread (secreted dependent), both of which occur in the myriad of infection types.

**Fig 6:**
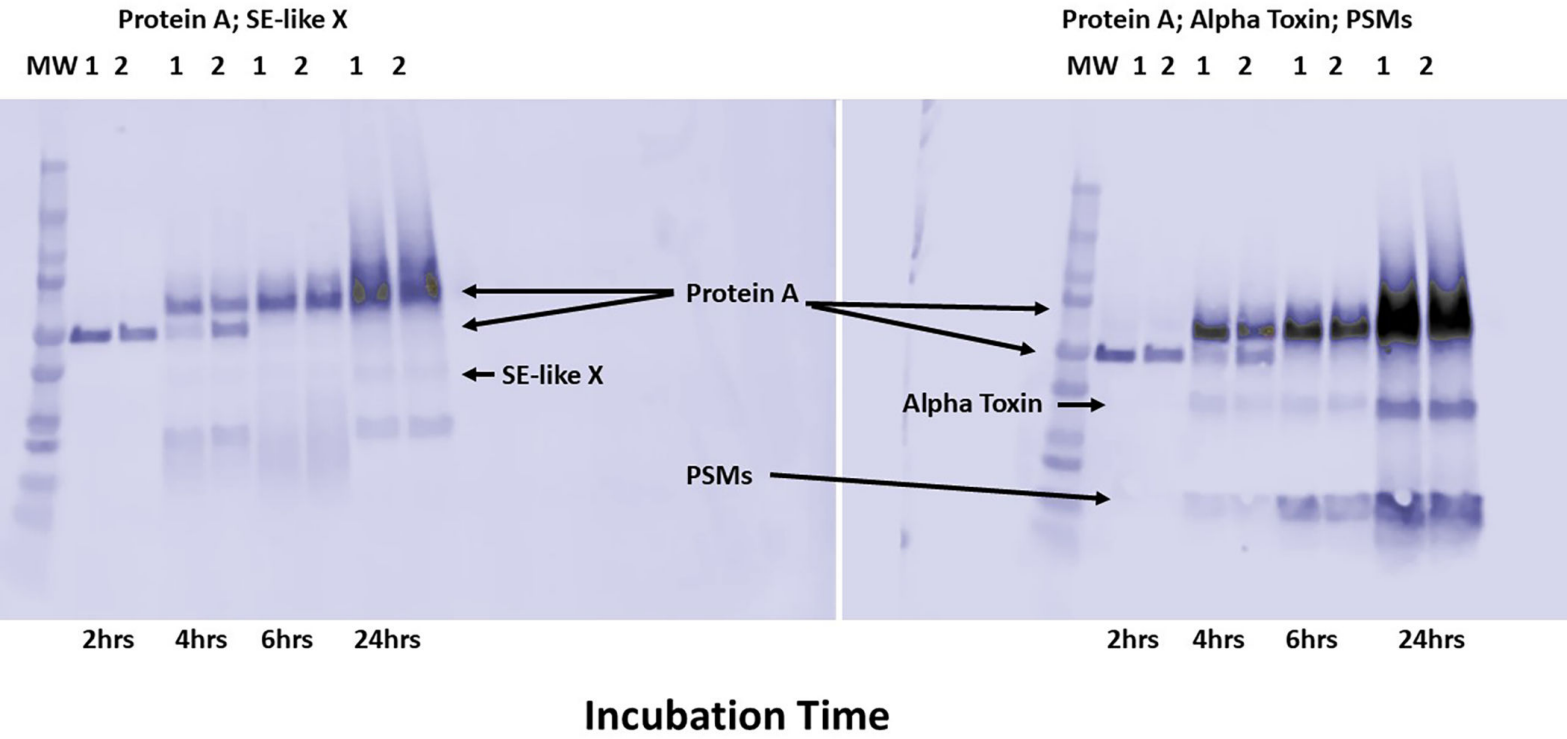
Western immunoblots of HP1 and HP2 (1 and 2) hyper-pink (on mannitol salt agar plates) *S. aureus* isolates. MW = molecular weight markers. HP1 and HP2 immunoblot probed with antibodies against the superantigen staphylococcal enterotoxin-like X (SE-like X; left blot). HP1 and HP2 immunoblot probed with antibodies against the cytotoxins α-toxin (alpha toxin) and phenol-soluble modulins (PSMs), mainly PSM-α3 (right blot). Protein A is a cell-surface virulence factor that occurs in multiple sizes by binding to the Fc region of IgG antibodies, irrespective of antigen specificity. There are up to four sizes of protein A. The organisms were grown for 24 h, with samples removed at the 2, 4, 6, and 24 h time points. The samples were clarified to remove bacterial cells. The inoculum size was approximately 10^7^ colony-forming units (CFUs)/mL. The stationary phase for organisms (10^10^ CFUs/mL) occurred prior to 4 h.

**Fig 7:**
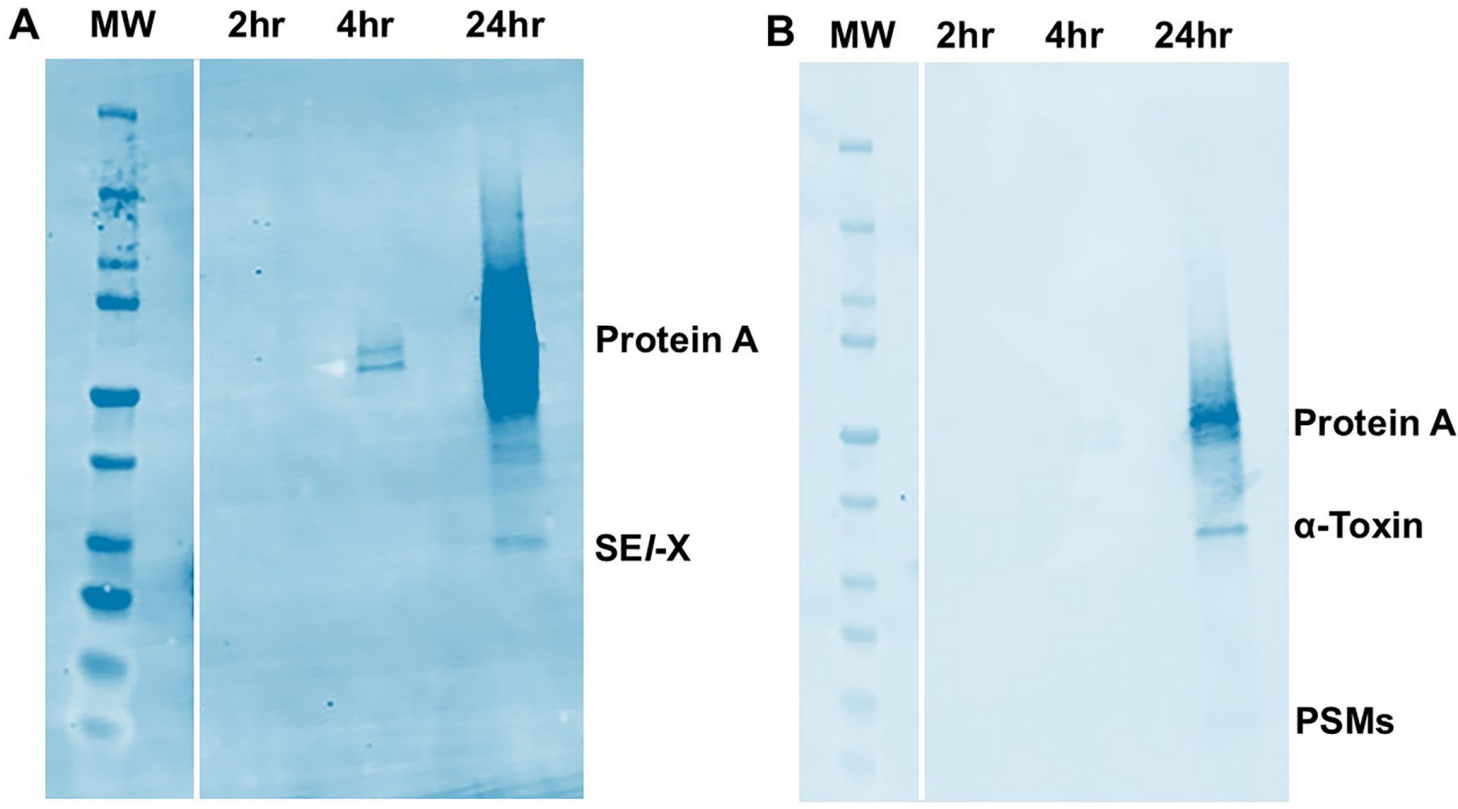
Western immunoblots of MNLE (USA300) non-hyper-pink *S. aureus* isolate. MW = molecular weight markers. (**A**) MNLE immunoblot probed with antibodies against the superantigen staphylococcal enterotoxin-like X (SE*l-*X). (**B**) MNLE immunoblot probed with antibodies against the cytotoxins α-toxin (alpha toxin) and phenol-soluble modulins (PSMs), mainly PSM-α3. Protein A is a cell-surface virulence factor that occurs in multiple sizes by binding to the Fc region of IgG antibodies, irrespective of antigen specificity. There are up to four sizes of protein A. The organisms were grown for 24 h, with samples removed at the 2, 4, and 24 h time points. The samples were clarified to remove bacterial cells. The inoculum size was approximately 10^7^ colony-forming units (CFUs)/mL. The stationary phase for the organisms (7.0 × 10^9^ CFUs/mL) occurred prior to 4 h.

With the above hypothesis in mind, we tested HP1 and HP2 for protein A and exotoxin production as a function of growth phase. Our prediction was that, since the mannitol utilization pathway could not be expressed, this change would affect medium pH and, consequently, exotoxin production, possibly extending exotoxin production into the late stationary phase. We thus grew both HP1 and HP2 (Fig. 6), along with a methicillin-sensitive USA300 organism (MNLE) as a control (Fig. 7), during both post-exponential (2 to 4 h) and late stationary phases (≥4 h), and evaluated the organisms for production of protein A and three secreted virulence factors. We observed exotoxin production by HP1 and HP2 (α-toxin, phenol-soluble modulin-α3, and SE*l*-X) in both the post-exponential phase and even more in the late stationary phase, in agreement with our hypothesis. However, the same secreted virulence factor production profile was observed for the USA300 strain MNLE (Fig. 7). The pH of the Todd Hewitt medium correlated with increased exotoxin production into stationary phase, as the pH remained basic during the entire growth period (>7.5). Also, increased production of protein A (a cell-surface factor) extended beyond exponential phase. HP1 and HP2, along with the control USA300 strain MNLE, produced more protein A in post-exponential and late stationary phases than in the exponential phase (Fig. 6). The expected multiple sizes of protein A were seen.

**Fig 8:**
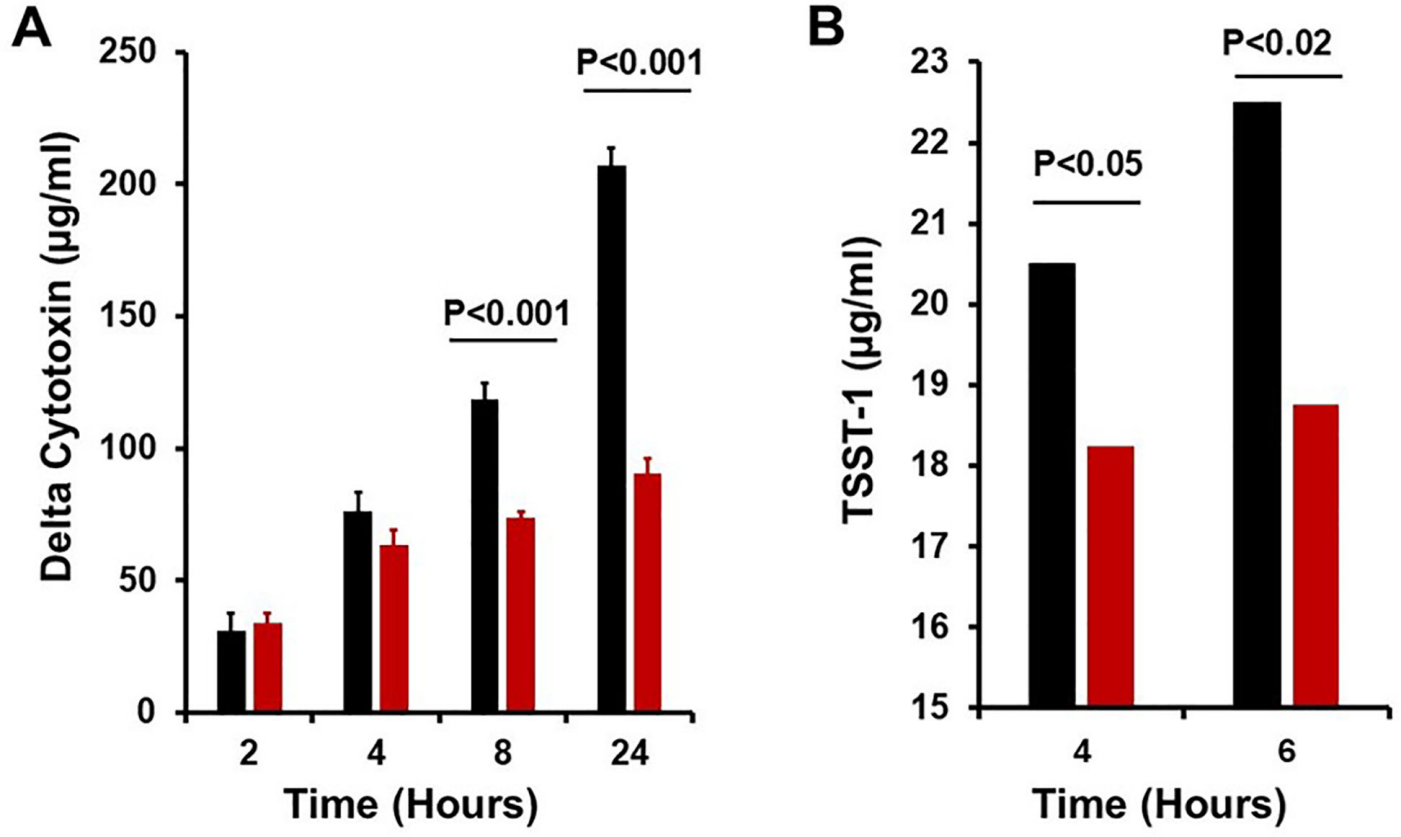
Effect on exotoxin production of maintaining pH at 8.0 compared to not maintaining pH in *S. aureus* MN8. (**A**) Effect on production of δ-cytotoxin. (**B**) Effect on production of toxic shock syndrome toxin-1 (TSST-1). Black bars: pH maintained at 8.0. Red bars: pH not maintained at 8.0 (final pH 5.5). Starting inoculum was approximately 10^7^ colony-forming units (CFUs)/mL. The post-exponential phase was 2–4 h, and the stationary phase was ≥6 h. Post-exponential phase was approximately 5 × 10^8^ to 5 × 10^9^ CFUs/mL. Stationary phase was 7.0 × 10^9^ CFUs/mL.

The above data also suggest that it may be possible to grow a non-hyper-pink *S. aureus*, which normally acidifies mannitol salt agar, turning the medium yellow, while maintaining the pH at 8.0. This could lead to increased exotoxin production in late stationary phase compared to post-exponential phase. We thus grew *S. aureus* strain MN8 (which produces δ-toxin and TSST-1), either without pH maintenance or with the pH maintained at 8.0, and tested for production of the two exotoxins in post-exponential (4 h) and stationary phases (6–24 h). As long as it was measured, beginning in post-exponential phase and extending into late stationary phase, δ-cytotoxin levels continued to rise (Fig. 8A). The RNA from this cytotoxin is a component of the Agr global regulatory system, which is required for exotoxin production (11). The data suggest that the Agr system remained active into stationary phase when exotoxin production is usually stopped. TSST-1 production was demonstrated in the post-exponential phase (Fig. 8B), but the toxin was produced in even higher amounts in late stationary phase, when exotoxin production usually ceases.

**Fig 9:**
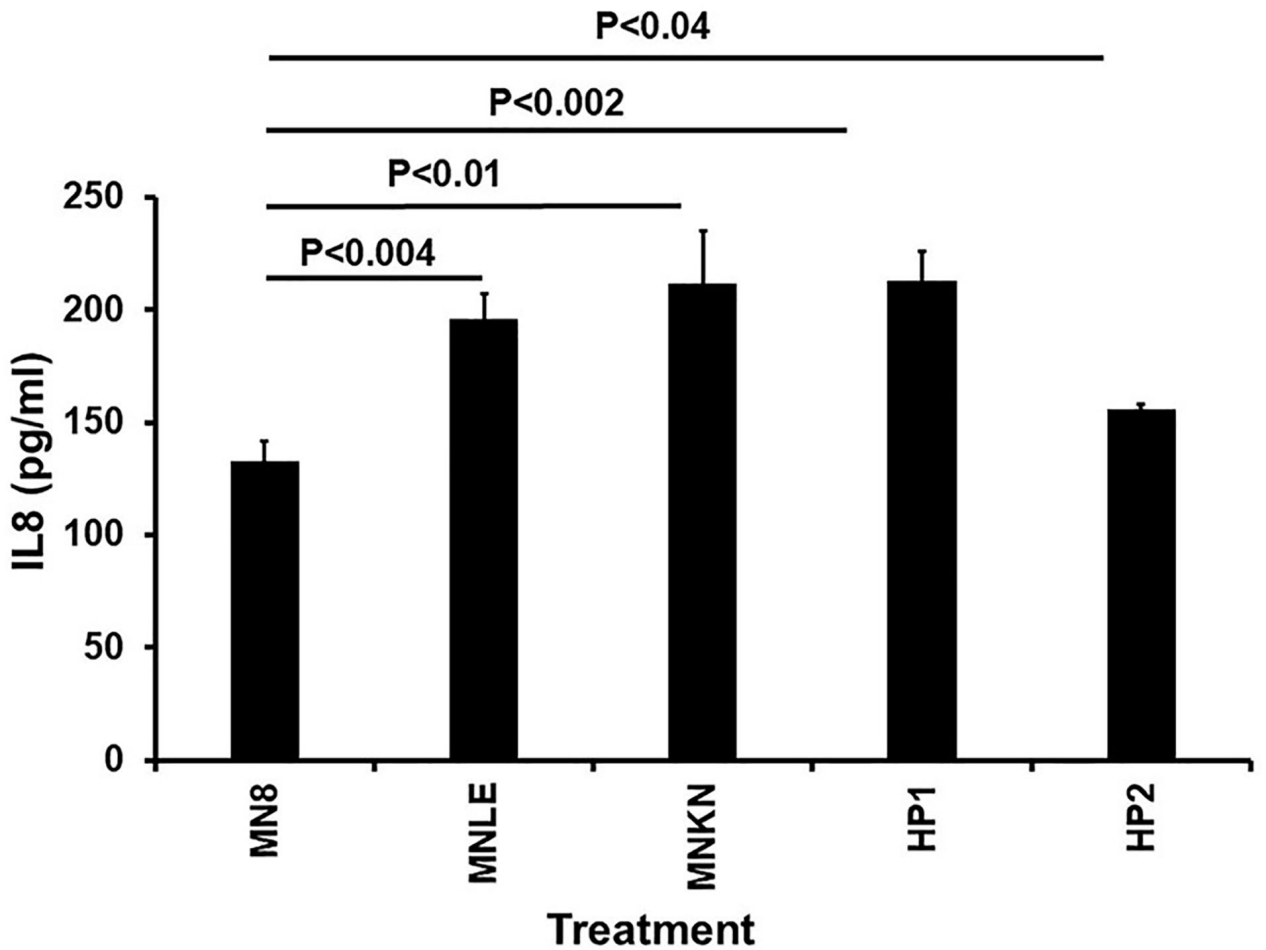
Effect of cell-free, stationary-phase culture fluids of *S. aureus* MN8 (USA200; mucosal; menstrual toxic shock syndrome [TSS]), MNLE (USA300; methicillin-sensitive; skin and hemorrhagic pneumonia), MNKN (USA400; skin and purpura fulminans), HP1, and HP2 on human vaginal epithelial cell production of pro-inflammatory IL-8. MN8 produces the major superantigen TSST-1 but produces only very small amounts of the cytotoxin α-toxin. MNKN produces the major superantigen staphylococcal enterotoxin C and high amounts of the cytotoxin α-toxin. MNLE, HP1, and HP2 produce the major superantigen SE*l*-X and high amounts of the cytotoxin α-toxin. *P* values compare the means of MN8 stimulation to means of the other four isolates.

The final set of studies evaluated the potentially harmful pro-inflammatory properties of HP1 and HP2 compared to MN8 (USA200), MNLE (USA300), and MNKN (USA400) (Fig. 9). Our prior studies have suggested that most skin and mucous membrane pathogens induce harmful chemokine production (IL-8 attraction of neutrophils; MIP-3α attraction of other components of the adaptive immune system) to facilitate disease causation (25–29). The latter three organisms were obtained from patients with vaginal mucosal menstrual TSS (MN8) (30), skin infections spreading to hemorrhagic pneumonia (MNLE), or purpura fulminans (MNKN) (31). It would be expected that *S. aureus* MN8 would not be as strongly pro-inflammatory as the other organisms, since MN8 has a mutation in the α-toxin (cytotoxin) structural gene that reduces pro-inflammatory α-toxin production by 50-fold compared to other *S. aureus* clonal groups (32). MN8 produces pro-inflammatory TSST-1. MNLE, HP1, and HP2 produce wild-type amounts of both pro-inflammatory α-toxin and SE*l-*X. MNKN produces wild-type amounts of both pro-inflammatory α-toxin and SEC. We compared the production of the representative chemokine IL-8 by human vaginal epithelial cells after 6 h of exposure to cell-free, stationary-phase cultures of the five pathogens. MNLE, MNKN, HP1, and HP2 all stimulated significantly more IL-8 than MN8. Dilution of all five culture fluids by 10- or 100-fold resulted in approximately proportional reduction in IL-8 production.

Taken together, our data suggest that the hyper-pink phenotype is a *S. aureus* clone, presently emerging. This strain makes growth media more basic (hyper-pink), allowing exotoxin production to increase well into the stationary phase of growth. The consequences of this phenotype are twofold: (i) potential confusion on mannitol salt agar plates with coagulase-negative opportunistic pathogens, and (ii) the ability to contribute to human diseases.

## DISCUSSION

*Staphylococcus aureus* is one of the most important pathogens causing human infections, commonly originating on mucous membranes and skin. Mucosal isolates usually produce only low amounts of the highly pro-inflammatory cytotoxin α-toxin, whereas skin isolates produce usual wild-type amounts of α-toxin (32). *S. aureus* infections include those leading to or contributing to TSS (4, 20), atopic dermatitis (33), cystic fibrosis (34), and diabetic foot ulcers (35), sepsis with infective endocarditis (36), osteomyelitis (37), and pneumonia (5, 8, 38). Both cell-surface and secreted virulence factors are important in disease causation (39, 40). Recsei et al. first showed that these virulence factors are differentially regulated by an operon known as Agr (11, 41, 42). These studies were followed by the identification of multiple other global regulators of virulence factor production (12, 16). These studies have all shown that cell-surface virulence factors are most often produced during exponential phase of growth, whereas secreted virulence factors (exotoxins and exoenzymes) are produced in a short period of time in post-exponential phase, with virulence factors not usually being produced in stationary phase.

A major method to isolate *S. aureus* is primary isolation on mannitol salt agar, where *S. aureus* turns the medium bright yellow (acidification) after 24–48 h of growth at 37°C, followed by tests for catalase and coagulase, both of which should be positive. The Schlievert laboratory has evaluated thousands of *S. aureus* isolates since 1980, many of which were cultured on mannitol salt agar. Up until 2020, we isolated only *S. aureus* that turned mannitol salt agar yellow. However, beginning in 2021, we began isolating *S. aureus* strains that did not turn mannitol salt yellow, but instead turned the medium bright pink (which we called hyper-pink). The strains have primarily oxidative metabolism on mannitol salt agar, resulting in basic pHs after 24–48 h of growth. These strains appear to have the same deletion mutation in the mannitol fermentation operon, indicating that they are likely to be clonal. The organisms were isolated from diverse areas across the United States, indicating the spread of the clone. The hyper-pink organisms appear most closely related to USA300 MRSA, but the hyper-pink organisms are methicillin-sensitive, similar to USA300 MNLE evaluated in this study. These organisms (HP1, HP2, and MNLE) are interesting in that they produce both cell-surface and secreted virulence factors well into stationary phase. This is a novel finding in our study.

The observation that we saw none of these hyper-pink organisms prior to 2020 and are now seeing them more frequently suggests that this hyper-pink clone is emerging. Historically, new clones arise in the U.S. population every 10 years (1–3), interestingly peaking in the fifth year of the decade and then declining. The two exceptions to this observation were USA200 and USA300 *S. aureus*, producing TSST-1 and SE*l-*X, respectively. The USA200 organisms emerged in 1971 and peaked in 1975, but the organism did not fall into the background (1–3). We think this emergence and continued presence depended on the production of the superantigen TSST-1 (4, 20), and thus conferred a selective advantage in humans. USA300 *S. aureus* emerged into the 2000s and continued into the next decade. These organisms produce SE*l*-X, which is important in the development of hemorrhagic pneumonia (18). TSST-1 and SE*l-*X are the only two superantigens that belong to structural group 1 (40). One or the other of these same two superantigens is produced by all pathogenic *S. aureus* strains, with most strains producing TSST-1 or SE*l-*X, but not both (19, 21). USA300 strains are now falling off in isolation frequency. It is also important to note that approximately 10% of *S. aureus* isolates obtained after 2020 exhibited the hyper-pink phenotype. This is approximately the same number of emergent USA200, USA400, and USA300 strains in the general *S. aureus* community.

We do not know why hyper-pink *S. aureus* and MNLE produce both cell-surface and secreted virulence factors well into the stationary phase. However, we have shown that the same phenomenon is observed if the medium pH is maintained as basic. This suggests that these organisms have active global regulators of virulence factors into stationary phase. We have suggested that the top regulator of the many global regulators in *S. aureus* (16) is the indirect oxygen-sensing TCS SrrA/B (12, 13). Secreted virulence factors are only produced in the presence of oxygen (13, 14). For example, the presence of oxygen trapped in tampons appears to be the reason for their association with menstrual TSS caused by the superantigen TSST-1 (14, 43). Downstream of SrrA/B is the quorum sensing system Agr, including the TCS AgrA/C (12). The direct Agr component that signals production of secreted virulence factors is the mRNA that also encodes δ-toxin. Although it has previously been shown that cell-surface and secreted virulence factors are differentially regulated by Agr, we observed in this study that δ-toxin mRNA continues to drive δ-toxin production into late stationary phase, so long as the pH is maintained as basic.

We have also shown that the hyper-pink organisms HP1 and HP2 were pro-inflammatory after interaction with human vaginal epithelial cells, comparable to that seen with USA300 and USA400 skin isolates that cause serious human diseases. We studied IL-8 as representative of pro-inflammatory chemokines. IL-8 attracts neutrophils to areas of infection. Our extensive studies in this area suggest that many mucosal and skin pathogens interact with CD40 and other receptors, for example, gp130, to facilitate harmful inflammation and promote subsequent disease (25–27, 44–46). Our findings with studies of IL-8 production are paralleled by another chemokine, MIP-3α, which attracts other components of the immune system. In this study, we compared IL-8 production by HP1, HP2, MNKN, and MNLE to the lesser production by MN8. *S. aureus* is a vaginal, menstrual TSS isolate with a mutation in the structural gene for α-toxin (32), a highly pro-inflammatory toxin produced in high levels by skin strains. Our findings indicate that the hyper-pink organisms are equally pro-inflammatory as MNKN and MNLE.

In sum, in this study, we describe the emergence of a novel clone of *S. aureus*, a clone that may be similarly virulent as other clonal groups, and a clone that may be confused with coagulase-negative, opportunistic staphylococci on mannitol salt agar. Other than identification of this new *S. aureus*, where the mannitol salt agar plates may be discarded with no obvious *S. aureus*, the organisms are expected to be virulent, contributing to a myriad of infections. A final consideration is that human tissues are not normally acidic; for example, the bloodstream, pulmonary tissue, and vagina during menstruation are buffered to pH 7.2. Oxygen is also present in all of these situations. Under such conditions, the presence of the mannitol use operon may not offer a selective advantage.

## MATERIALS AND METHODS

### Bacteria

The pre-2021 clinical isolates of *S. aureus* included combinations of 200 of USA200 (including MN8), USA300 (including MNLE), and USA400 (including MNKN) from menstrual TSS, hemorrhagic pneumonia, and purpura fulminans. These isolates were submitted to the Schlievert laboratory for superantigen testing, beginning in 1980. Also included were 122 clinical isolates from patients with moderate to severe atopic dermatitis. Finally, the hyper-pink clinical isolates, including HP1 and HP2, most often referred to in this study and collected post-2020, were cultured from patients with moderate to severe atopic dermatitis (33). Five additional hyper-pink organisms were studied. For some studies, eight non-hyper-pink organisms were evaluated. Strain MN8 was a USA200 menstrual TSS isolate from 1980 (14). Strain MNWH was a USA200 MRSA menstrual TSS isolate. Strains MW2, MNAS, MNKN (31), and MNHO (47) were USA400 MRSA isolates from patients with hemorrhagic pneumonia and/or purpura fulminans. MNLE was a USA300 methicillin-sensitive hemorrhagic pneumonia isolate. MNLAC was a USA300 MRSA. All organisms, of low passage, were stored as −80°C stocks. The organisms were cultured in commercially available mannitol salt agar and Todd Hewitt broth (Difco, Detroit, MI). All organisms were verified as coagulase-positive as determined by slide agglutination for clumping factor.

The Schlievert laboratory has tested a large number of *S. aureus* clinical isolates from patients with TSS and hemorrhagic pneumonia. Many of these from 1980 to 2020 have been grown on mannitol salt agar, and none had the hyper-pink phenotype.

### Human vaginal epithelial cells

Immortalized human vaginal epithelial cells were cultured in keratinocyte, serum-free medium to confluence in 96-well flat-bottom tissue culture plates (44, 48). At that time, cell-free, sterile stationary-phase culture fluids of various *S. aureus* strains were added for 6 h in quadruplicate. Subsequently, IL-8 production by the human vaginal epithelial cells was quantified by a Quantikine ELISA kit purchased from R&D Systems (Minneapolis, MN) and compared with the control IL-8 provided with the kit. It is important to note that the human vaginal epithelial cells do not have the Toll-like receptor 4 and are thus not responsive to potentially contaminant lipopolysaccharide.

### Nucleotide sequencing and PCR

HP1 and HP2 *S. aureus* DNA were isolated (33), and the DNA was submitted to the SeqCenter (Pittsburgh, PA). The sequences were analyzed by NCBI programs for the presence of global regulatory DNA elements and genes for virulence factors. Sequence ID numbers at NCBI are 3018670 and 3018667. DNA from HP1 to HP7 was isolated similarly and tested by PCR for deletion of components of the mannitol utilization pathway. Primers were as described previously (10).

### SDS-PAGE and western immunoblotting

HP1, HP2, and MNLE were cultured in Todd Hewitt broths for up to 24 h. MNLE was chosen as the control isolate because the organism produced the superantigen SE*l*-X, but not other superantigens, similar to HP1 and HP2. Also, all three isolates produced both α-toxin and protein A. Colony-forming units (CFUs)/mL were determined at selected time points to assess exponential, post-exponential, and stationary phases. Supernatant fluids from the organisms were treated with four volumes of absolute ethanol to precipitate proteins, including cytotoxins, superantigens, and protein A (49). The precipitates were collected (14,000 × *g*, 5 min) and resolubilized at 10× in distilled water. Insoluble material was removed by centrifugation (14,000 × *g*, 5 min). SDA-PAGE was performed with pre-cast gels (49). After electrophoresis, the proteins in the gels were transferred to PVDF membranes and probed with monospecific hyperimmune rabbit antibodies against SE*l-*X or TSST-1 (superantigens) or hyperimmune rabbit antibodies that recognize both α-toxin and PSM-α3 (cytotoxins) or δ-toxin. The immunoblots were then developed with conjugate antibodies against rabbit IgG, scanned on a LiCor Odyssey, and density of bands was determined using NIH ImageJ for quantification.

### pH experiments

Bacteria were cultured for designated times in Todd Hewitt broths (pH 7.5) at 37°C with shaking at 200 RPM. The pH was adjusted to 8.0 using 10 M NaOH. A pH of 8.0 with aeration (200 RPM shaking) provides an excellent condition for secreted virulence factor production (14). The pH was either maintained at 8.0 using 10 M NaOH, or not maintained during growth of *S. aureus* MN8. pH at selected times was measured with a standard pH meter.

In some experiments, lawns of organisms were cultured on mannitol salt agar plates for up to 48 h. Then, the agarose with grown lawns of bacteria was immersed in 10 mL of distilled water. The mixtures were stirred for 2 min, and then, pH was measured with a standard pH meter.

### Statistics

Means ± standard deviations were determined. Differences in means were assessed using unpaired Student’s *t*-test.
